# Supplementary material for: Investigating immune amnesia after measles virus infection in two West African countries: A study protocol
Source: PLoS One. 2025 Jun 26;20(6):e0314828. doi: 10.1371/journal.pone.0314828 (PMC12200839; doi:10.1371/journal.pone.0314828)
Supplement: S1 File — (DOCX) [file pone.0314828.s001.docx]

**Investigation of Immune Amnesia Following Measles Infection in Select African Regions**

**Protocol #:** 2023-CCRB-0902

**Version** 3.0

**Date:** 11 March 2024

In-country Study Teams

Country Principal Investigators (PIs), study teams, and local Institutional Review Boards (IRBs)/Ethics Committees (ECs) can be found in each country’s site-specific appendix (SSA).

Other Investigators

Drs. Diane Griffin and Jessica Rubens, Johns Hopkins University, are measles scientific expert consultants.

Sponsor: National Institute of Allergy and Infectious Diseases (NIAID)

NIAID Scientific Advisors

Dr. Katy Shaw - Saliba - protocol lead

Dr. Ray Chen, Dr. Sally Hunsberger, Dr. Renee Ridzon, Dr. Jamila Aboulhab and Dr. Karine Fouth Tchos -protocol advisors

Dr. Caeul Lim - laboratory advisor

Ms. Mary Smolskis - operations advisor

Ms. Esther Akpa - operations lead

NIAID staff will not obtain identifiable personal information about the participants and will be responsible for studying, interpreting, or analyzing coded (linked) data or specimens for research analysis.

| Rabies Vaccine Provided by | Sanofi Pasteur  14 Espace Henry Vallée,  Lyon, France |
| --- | --- |

**TABLE OF CONTENTS**

[STATEMENT OF COMPLIANCE 4](#_Toc161055450)

[1 PROTOCOL SUMMARY 5](#_Toc161055451)

[1.1 Synopsis 5](#_Toc161055452)

[1.2 Schema 7](#_Toc161055453)

[1.2.1 Cohorts 7](#_Toc161055454)

[1.2.2 Timing of visits 8](#_Toc161055455)

[1.3 Schedule of Activities 9](#_Toc161055456)

[2 INTRODUCTION 12](#_Toc161055457)

[2.1 Study Rationale 12](#_Toc161055458)

[2.2 Background 12](#_Toc161055459)

[2.3 Risk/Benefit Assessment 13](#_Toc161055460)

[2.3.1 Known Potential Risks 13](#_Toc161055461)

[2.3.2 Known Potential Benefits 14](#_Toc161055462)

[2.3.3 Assessment of Potential Risks and Benefits 15](#_Toc161055463)

[3 STUDY OBJECTIVES 16](#_Toc161055464)

[4 STUDY DESIGN 17](#_Toc161055465)

[4.1 Overall Design 17](#_Toc161055466)

[4.2 Study Sites 19](#_Toc161055467)

[4.3 Concomitant Therapy 19](#_Toc161055468)

[5 STUDY POPULATION 19](#_Toc161055469)

[5.1 Inclusion Criteria 19](#_Toc161055470)

[5.2 Exclusion Criteria 20](#_Toc161055471)

[5.3 Inclusion of Vulnerable Participants 20](#_Toc161055472)

[5.4 Inclusion of Pregnant Women, Fetuses, or Neonates 20](#_Toc161055473)

[5.5 Screen Failures 21](#_Toc161055474)

[5.6 Strategies for Recruitment and Retention 21](#_Toc161055475)

[5.6.1 Costs 21](#_Toc161055476)

[5.6.2 Compensation 21](#_Toc161055477)

[6 PARTICIPANT DISCONTINUATION/WITHDRAWAL 21](#_Toc161055478)

[6.1 Participant Discontinuation/Withdrawal from the Study 21](#_Toc161055479)

[6.2 Lost to Follow-up 21](#_Toc161055480)

[7 STUDY ASSESSMENTS AND PROCEDURES 22](#_Toc161055481)

[7.1 Screening Procedures 22](#_Toc161055482)

[7.1.1 Activities Performed Prior to Obtaining Informed Consent 22](#_Toc161055483)

[7.1.2 Activities Performed After a Consent for Screening Has Been Signed 22](#_Toc161055484)

[7.2 Description of Study Procedures 23](#_Toc161055485)

[7.3 Biospecimen Evaluations and Correlative Studies for Research 24](#_Toc161055486)

[7.4 Management/Return of Results 24](#_Toc161055487)

[7.5 Adverse Events 25](#_Toc161055488)

[7.5.1 Definitions 25](#_Toc161055489)

[7.6 Classification of an Adverse Event 26](#_Toc161055490)

[7.6.1 Severity of Event 26](#_Toc161055491)

[7.6.2 Relationship to Study Procedures 26](#_Toc161055492)

[7.6.3 Type and Duration of the Follow-up of Participants After AEs 26](#_Toc161055493)

[7.7 Reporting 26](#_Toc161055494)

[8 STATISTICAL CONSIDERATIONS 27](#_Toc161055495)

[8.1 Statistical Hypothesis 27](#_Toc161055496)

[8.2 Sample Size Determination 27](#_Toc161055497)

[8.3 Feasibility Assessment 29](#_Toc161055498)

[8.4 Population for Analysis 29](#_Toc161055499)

[8.5 Statistical Analyses 29](#_Toc161055500)

[8.5.1 General Approach 29](#_Toc161055501)

[8.5.2 Analysis of Primary Endpoints 30](#_Toc161055502)

[8.5.3 Analysis of Secondary Endpoints 30](#_Toc161055503)

[9 REGULATORY AND OPERATIONAL CONSIDERATIONS 30](#_Toc161055504)

[9.1 Informed Consent Process 30](#_Toc161055505)

[9.1.1 Consent/Assent Procedures and Documentation 30](#_Toc161055506)

[9.2 Study Discontinuation and Closure 31](#_Toc161055507)

[9.3 Future Use of Stored Specimens and Data 31](#_Toc161055508)

[9.4 Confidentiality and Privacy 32](#_Toc161055509)

[9.5 Study Monitoring 32](#_Toc161055510)

[9.5.1 Data and Safety Monitoring 32](#_Toc161055511)

[9.5.2 Clinical Monitoring 33](#_Toc161055512)

[9.6 Quality Assurance and Quality Control 33](#_Toc161055513)

[9.7 Data Handling and Record Keeping 33](#_Toc161055514)

[9.7.1 Data Collection and Management Responsibilities 33](#_Toc161055515)

[9.7.2 Study Records Retention 34](#_Toc161055516)

[9.8 Protocol Deviations and Non-Compliance 34](#_Toc161055517)

[9.9 Reporting to the NIAID Clinical Director 34](#_Toc161055518)

[9.10 Publication and Data Sharing Policy 35](#_Toc161055519)

[9.10.1 Human Data Sharing Plan 35](#_Toc161055520)

[9.10.2 Genomic Data Sharing Compliance 35](#_Toc161055521)

[9.11 Collaborative Agreements 35](#_Toc161055522)

[9.11.1 Agreement Type 35](#_Toc161055523)

[9.12 Conflict of Interest Policy 35](#_Toc161055524)

[10 ABBREVIATIONS 35](#_Toc161055525)

[11 REFERENCES 37](#_Toc161055526)

## STATEMENT OF COMPLIANCE

This study will be conducted in compliance with the International Council for Harmonisation of Technical Requirements for Pharmaceuticals for Human Use (ICH) E6(R2) Good Clinical Practice (GCP) guidelines and the applicable regulatory requirements at participating institutions.

# PROTOCOL SUMMARY

## Synopsis

| **Title:** | Investigation of Immune Amnesia Following Measles Infection in Select African Regions |
| --- | --- |
| **Study Description:** | This is a prospective, observational, longitudinal study to be conducted in West Africa to investigate the effects of measles virus (MeV) infection on pre-existing immunity, vaccine response, and susceptibility to subsequent illness. Eligible children will be enrolled into 1 of 2 arms: acute MeV infection (cases) or no acute MeV infection (controls) as assessed using upper respiratory specimens and blood samples. Blood samples will be collected at Screening/Enrollment (Day 0), optional repeat IgM serology for inconclusive or negative Day 0 results (Day 7-10), and follow-up visits on Day 14, Week 13, and Week 52. The blood samples will be tested for humoral and cellular immune responses to endemic pathogens to determine changes in antibody diversity and antibody secreting cells (ASCs). All children in each arm will receive rabies vaccination (standard 3-dose series given as pre-exposure prophylaxis [PrEP]), with the first dose randomized to either Week 8 or Week 47 after enrollment. Biological samples will be collected after vaccination to assess if the immune stimulus (rabies vaccine) response differs: 1) between children with and without MeV infection, and 2) based on the timing of the receipt of the rabies vaccine. The study team will collect, to the extent possible, information on healthcare encounters for evaluation and treatment of illness during the year-long follow-up to determine if there is a difference in the number of encounters by study group. |
| **Objectives:** | Primary Objectives   1. To determine if MeV infection induces a loss of pre-existing immunity (immune amnesia) to endemic pathogens at Week 13 after baseline in children in select African regions. 2. To determine the effect of MeV infection on immune response to a controlled immune stimulus (rabies vaccination) at early and late timepoints post-infection.   Secondary Objectives   1. To determine if there is an increase in the healthcare system encounters in the year following enrollment in children with recent MeV infection compared to those without recent MeV infection. 2. To determine if MeV infection induces a loss of pre-existing immunity (immune amnesia) to endemic pathogens at Week 52 after baseline in children in select African regions.   Exploratory Objectives   1. To assess the appearance and continued production of B and T cells, especially ASCs from peripheral blood mononuclear cells (PBMCs) in circulation following MeV infection in children 1-15 years old in a subset of participants depending on availability of collected blood samples. 2. To attempt to identify the etiology of illness in children who present with signs and symptoms consistent with measles and are screened but not enrolled due to negative measles polymerase chain reaction (PCR) and immunoglobulin M (IgM). 3. To identify the genotypes of MeV collected in the study. 4. To characterize the phenotype of the rash and/or Koplik spots in children who present with signs and symptoms consistent with measles who test positive for measles (PCR and/or IgM positive) and who test negative for measles (PCR and IgM negative). |
| **Accrual Ceiling:** | A maximum of 500 across all study sites. |
| **Study Population:** | We will enroll approximately 256 children aged 1-15 years into 1 of 2 study groups:   - Group 1 (Cases): acute MeV infection (n=128) with approximately n=64 age 1-5 years and approximately n=64 age 6-15 years, and each age group randomized 1:1 to receive rabies vaccine at Week 8 or Week 47 after enrollment - Group 2 (Controls): no acute MeV infection (n=128) with approximately n=64 age 1-5 years and approximately n=64 age 6-15 years, and each age group randomized 1:1 to receive rabies vaccine at Week 8 or Week 47 after enrollment |
| **Description of Sites/Facilities Enrolling Participants:** | The study is expected to take place in a subset of the NIAID Division of Clinical Research (DCR) Special Projects sites and any additional site(s) that may be identified. |
| **Participant Follow up:** | 12 months. |
| **Study Duration:** | Up to 48 months. |

## Schema

### Cohorts

|  |
| --- |

Figure 1: Study cohorts

### Timing of visits


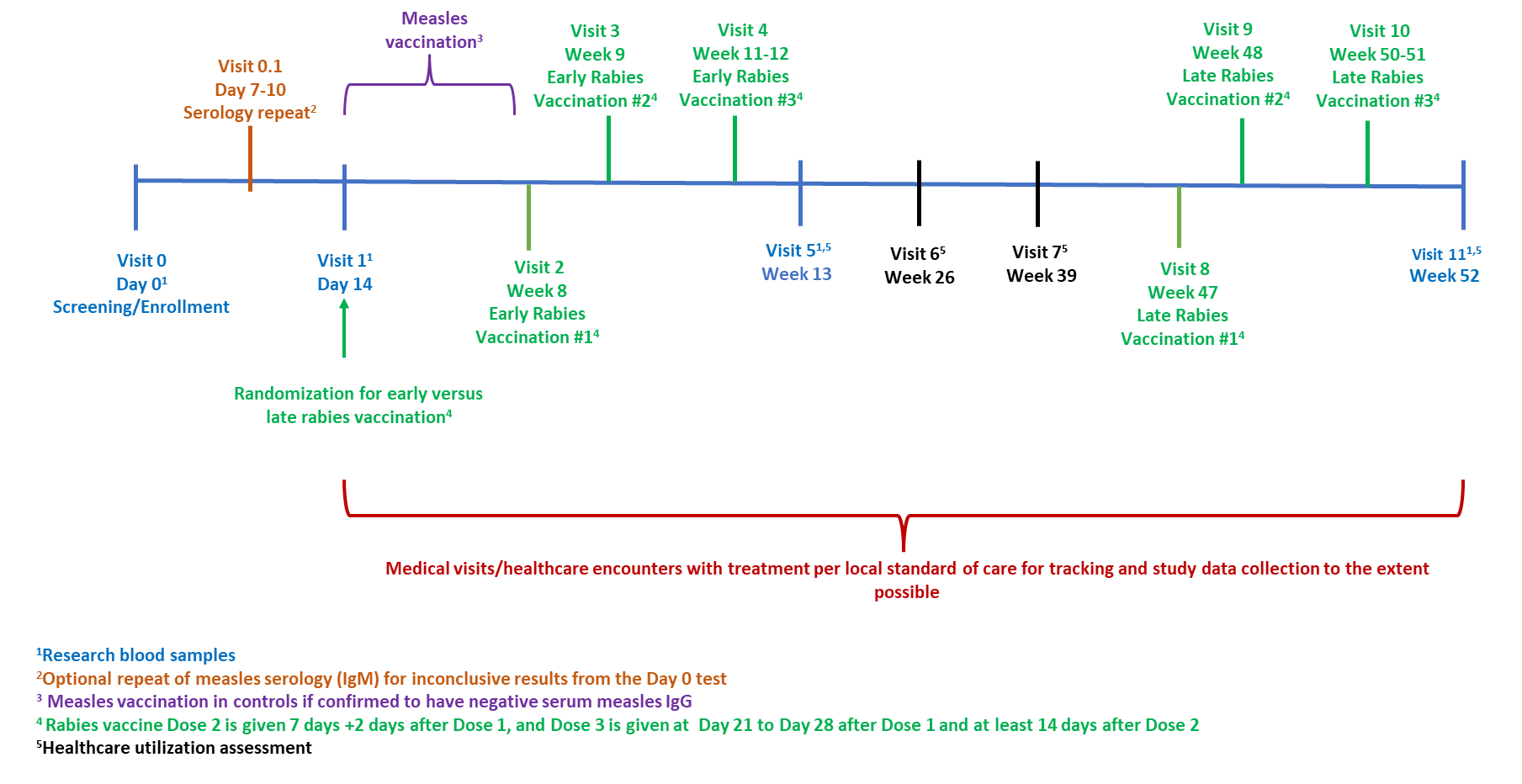


Figure 2: Timing of visits with associated procedures

## Schedule of Activities

Table 1. Schedule of study evaluations and procedures.

|  | **Visit 0** | **Visit 0.1** | **Visit 1** | **Visit 2^a^** | **Visit 3^a^** | **Visit 4^a^** | **Visit 5** | **Visit 6** | **Visit 7** | **Visit 8^b^** | **Visit 9^b^** | **Visit 10^b^** | **Visit 11** |
| --- | --- | --- | --- | --- | --- | --- | --- | --- | --- | --- | --- | --- | --- |
| **Evaluation/**  **Procedure** | **Screening/ Enrollment^c^ Blood Draw** | **Serology repeat (Retest for measles IgM)** | **Randomization**  **Blood Draw** | **Early rabies vaccine Dose 1** | **Early rabies vaccine Dose 2** | **Early rabies vaccine Dose 3** | **Blood draw**  **Healthcare Ques** | **Healthcare Ques** | **Healthcare Ques** | **Late rabies vaccine Dose 1** | **Late rabies vaccine Dose 2** | **Late rabies vaccine Dose 3** | **Blood draw**  **Healthcare Ques** |
| **Timepoint**  **(Window)** | **Day 0^c^** | **Day 7-10^c^** | **Day 14^c^**  **(±2 days)** | **Week 8**  **(±3 days)** | **Week 9**  **(+2 days)** | **Week 11-12** | **Week 13**  **(+3 days)** | **Week 26**  **(±2 weeks)** | **Week 39**  **(±2 weeks)** | **Week 47**  **(±3 days)** | **Week 48**  **(+2 days)** | **Week 50-51** | **Week 52**  **(+3 days)** |
| Informed consent/assent | X |  |  |  |  |  |  |  |  |  |  |  |  |
| Inclusion/ Exclusion^c^ | X | | |  |  |  |  |  |  |  |  |  |  |
| Vital signs^d^ | X |  | X | X^d^ | X^d^ | X^d^ | X | X | X | X^d^ | X^d^ | X^d^ | X |
| MUAC^e^, height, weight | X |  |  |  |  |  |  |  |  |  |  |  | X |
| Demographics | X |  |  |  |  |  |  |  |  |  |  |  |  |
| Upper respiratory specimen (OP/NP swab) for measles PCR | X |  |  |  |  |  |  |  |  |  |  |  |  |
| HIV^f^ | X^g^ |  |  |  |  |  |  |  |  |  |  |  | X^f^ |
| Hemoglobin level^h^ | X |  | X |  |  |  | X |  |  |  |  |  | X |
| Urine pregnancy test^h^ | X^i^ |  | X | X^i^ | X^i^ | X^i^ | X |  |  | X^i^ | X^i^ | X^i^ | X |
| Measles IgM/IgG | X^g^ | X |  |  |  |  |  |  |  |  |  |  |  |
| Medical history | X |  |  |  |  |  | X |  |  |  |  |  | X |
| Concomitant medications | X |  | X | X | X | X | X | X | X | X | X | X | X |
| Physical examination | X |  | X |  |  |  | X | X | X | X |  |  | X |
| Optional photography (skin rash and Koplik spots)^j^ | X |  |  |  |  |  |  |  |  |  |  |  |  |
| Randomization to early or late rabies vaccine |  |  | X |  |  |  |  |  |  |  |  |  |  |
| Measles vaccine^k^ |  |  | X |  |  |  |  |  |  |  |  |  |  |
| AE assessment |  |  | X | X | X | X | X | X | X | X | X | X | X |
| Healthcare Utilization Questionnaire |  |  |  |  |  |  | X | X | X |  |  |  | X |
| Rabies vaccine |  |  |  | (X) | (X) | (X) |  |  |  | (X) | (X) | (X) |  |
| Research blood collection^l^ | X |  | X |  |  |  | X |  |  |  |  |  | X |
| Vitamin A | X^m^ |  |  |  |  |  |  |  |  |  |  |  |  |

Abbreviations: AE, adverse event; HIV, human immunodeficiency virus; Ig, immunoglobulin; MUAC, mid-upper arm circumference; NP, nasopharyngeal; OP, oropharyngeal; PCR, polymerase chain reaction.

(X) = Each participant will receive only one 3-dose series of rabies vaccine according to randomization to early group (vaccination at Visits 2, 3, and 4) or late group (vaccination at Visits 8, 9, and 10).

^a^ EARLY rabies vaccination: Only participants randomized to EARLY rabies vaccine attend Visits 2, 3, 4. Visit 2 occurs 8 weeks after Day 0; Visit 3 occurs 7 days after Visit 2; Visit 4 occurs 21 to 28 days after Visit 2 AND at least 14 days after Visit 3.

^b^ LATE rabies vaccination: Only participants randomized to LATE rabies vaccine attend Visits 8, 9, 10. Visit 8 occurs 47 weeks after Day 0; Visit 9 occurs 7 days after Visit 8; Visit 10 occurs 21 to 28 days after Visit 8 AND at least 14 days after Visit 9.

^c^ Day 0 is defined as the day screening occurs and the first research blood sample is collected. Inclusion/exclusion criteria evaluation for enrollment will be completed when PCR and measles IgG/IgM and HIV results are available, generally by Day 14. In cases where the clinical presentation is consistent with measles but the Day 0 tests (measles PCR and measles IgM) are negative or equivocal, an optional retest of the measles IgM can be done between Days 7 and 10. The guardian/parent will be informed of the need for a retest and asked to bring the child back to the clinic. Blood will be collected via finger stick for the retest and results will be available by Day 14. All potential participants (or guardian/parent) will be informed of eligibility status by a designated study staff by phone, if possible, prior to the Day 14 scheduled study visit or at the Day 14 scheduled study visit. Ineligible participants may be invited to the site to discuss test results and referral for measles vaccination if serum IgG and IgM are both negative.

^d^ Vital signs include temperature, heart rate, and respiratory rate; may be obtained prior to rabies vaccine administration, if clinically indicated.

^e^ MUAC in children 12 to 59 months old.

^f^ HIV testing for children younger than 24 months will be performed via PCR and repeated with Rapid Diagnostic Test (RDT) at end of study to adhere to national guidelines; HIV RDT will be performed at screening for participants 24 months and older and will not be repeated at end of study.

^g^ HIV and measles serum IgM/IgG results must be reviewed for eligibility confirmation prior to Day 14.

^h^ Blood will be collected for hemoglobin test via finger and/or heel stick. Hemoglobin and urine pregnancy rapid test results must be reviewed prior to venous blood sample collection.

^i^ Females of child-bearing potential must have a negative pregnancy test prior to enrollment and each rabies vaccine administration.

^j^ Optional photography of the skin rash and/or Koplik spots for documenting the phenotype of the signs of the potential cases.

^k^ Measles vaccination to be offered to ineligible participants and administered to eligible control participants (through the study or referral to Ministry of Health vaccination program) if serum and PCR measles IgG and IgM are both negative.

^l^ 5 mL is the maximum volume that will be obtained at each blood draw.

^m^ Vitamin A will be administered at D0 to all participants with clinical measles per WHO guidelines: 2 oral doses of 200,000 IU given 24 hours apart. The first dose will be administered at the study site and the second will be provided to the parent to be administered 24 hours later.

# INTRODUCTION

## Study Rationale

A phenomenon of depletion of pre-existing antibodies and increase in susceptibility to infections, known as “immune amnesia,” following MeV infection has been reported by several studies as described below (section 2.2). Ongoing recurrent outbreaks of measles in West Africa allow a more detailed evaluation of the pathophysiology of immune amnesia and how, it affects the incidence of subsequent infections.

In this study, we will assess whether MeV infection: 1) induces a loss of pre-existing immunity to pathogens prevalent in children in select African regions; 2) results in decreased immune responses to a controlled immune stimulus (rabies vaccination); and 3) is associated with an increase in healthcare system encounters in the year following MeV infection.

## Background

The continual measles outbreaks in West Africa underscore the importance of studies that will contribute to an understanding of the impact of MeV infection on the immune system and associated morbidity and mortality. Over the past decade, West Africa has experienced measles outbreaks each year. In Guinea, for example, the number of confirmed cases from January to June 2022 in 22 of 38 health districts was 2,299, 69% of which were in children aged 6-59 months.[[1](#_ENREF_1)] As another example, Mali recorded 218 cases in January 2022, and this is likely an underestimation.[[2](#_ENREF_2)] From January to October 2022, 7,927 suspected measles cases were reported from 9 of the 15 counties in Liberia; 93% (7,377) of these were confirmed through laboratory testing, clinically compatible with measles, and epidemiologically linked to other measles cases.[[3](#_ENREF_3)] These continued outbreaks have largely followed large-scale public health emergencies such as the Ebola epidemic and the current COVID-19 pandemic, which has disrupted global routine childhood vaccine coverage.[[4-6](#_ENREF_4)] Thus, measles outbreaks can be expected to continue in the coming years.

MeV can be serious and is one of the leading causes of death for children globally.[[7-9](#_ENREF_7)] MeV is highly contagious, and high vaccine coverage (above 90%) is essential to curb transmission.[[7](#_ENREF_7), [10](#_ENREF_10)] In addition to the seriousness of acute disease, there is a lasting impact of MeV infection. While MeV infection results in a strong immune response that confers life-long immunity, there is a prolonged increase in susceptibility to other infections, known as “immune amnesia,” following MeV infection.[[11](#_ENREF_11)] This immune amnesia has been associated with a loss of memory B cells and reduced antibody repertoires from previous infections and vaccinations.[[12](#_ENREF_12), [13](#_ENREF_13)] Measles immune amnesia has also been associated with clinical effects. A number of studies have shown that measles vaccination is associated with larger reductions in morbidity and mortality than would be expected from that caused by acute measles disease alone.[[14-19](#_ENREF_14)] Other studies have identified increased rates of infections and/or hospitalizations after measles virus infection.[[18](#_ENREF_18), [20-23](#_ENREF_20)]

Previous studies characterizing MeV immune amnesia have primarily been centered around a European population and rhesus macaques.[[24](#_ENREF_24)] Given the current outbreaks of measles in West Africa and recurrence of infectious diseases in pediatric populations, there is interest in determining whether measles-induced immune amnesia occurs in these African regions where tuberculosis, parasitic diseases (such as malaria), and arthropod-borne viruses (such as yellow fever and dengue) are endemic, and if so, what the mechanisms are that drive immune amnesia. The hypothesis of this study is that acute MeV infection induces a loss of pre-existing immunity to pathogens prevalent in select African regions. Compared to those who do not have acute MeV infection, acute MeV infection will result in:

1. A loss of total antibody diversity from previous infections and/or vaccinations compared to baseline.
2. Altered immune response to a controlled immune stimulus (rabies vaccination).
3. Increased incidence of illnesses or healthcare encounters in the year following acute MeV infection.

## Risk/Benefit Assessment

### Known Potential Risks

Blood draws: Drawing blood may cause pain, bruising, lightheadedness, possible fainting, local discomfort, uncontrolled bleeding, and, rarely, infection at the site where the needle is inserted. The study staff will evaluate injuries related to research blood draw and provide appropriate first aid and referral for care as needed.

Urine collection: There is no risk from urine collection.

Upper respiratory specimen: The risks of obtaining a respiratory swab through the oropharynx/ nasopharynx include ear, nose and throat discomfort and pain, headache, rhinorrhea, anxiety, and gagging. In rare cases a mild and self-limiting epistaxis may occur after nasopharyngeal swabbing.

Rabies vaccination: Soreness, redness, swelling, or itching at the site of the injection, and headache, nausea, abdominal pain, muscle aches, or dizziness can happen after rabies vaccine. Hives, pain in the joints, or fever sometimes happen after each vaccine dose. People sometimes faint after medical procedures, including vaccination. As with any medicine, there is a very remote chance of a vaccine causing a severe allergic reaction, other serious injury, or death.

Measles vaccination: The main risks associated with measles vaccination are pain, redness, swelling or itching at the injection site. The measles vaccine has been associated with a very low risk of febrile seizures that are not associated with any long-term effects. Some people may experience swelling in the cheeks or neck. A temporary low platelet count can rarely occur, which can cause a bleeding disorder that usually goes away without treatment and is not life-threatening. In very rare cases, a person may have a severe allergic reaction. People sometimes faint after medical procedures, including vaccination. As with any medicine, there is a very remote chance of a vaccine causing a severe allergic reaction, other serious injury, or death. Anyone who has ever had a life-threatening allergic reaction to the antibiotic neomycin, or any other component of the measles vaccine should not receive the vaccine.

Vitamin A: When administered orally per World Health Organization (WHO) guidelines, sides effects are rare; mild symptoms typically develop within 24 hours and can include loose stools, headache, irritability, fever, nausea, and vomiting; these are reversible within 24 to 48 hours upon discontinuation of vitamin A.[[25](#_ENREF_25)] An acute intoxication typically occurs if more than 100 times the recommended dietary allowance of 10,000 IU is ingested; signs and symptoms occur within days to weeks of ingestion and typically include severe headache, blurred vision, nausea, dizziness, aching muscles, and coordination problems.[[26](#_ENREF_26)] In severe cases, cerebral spinal fluid pressure can increase, leading to drowsiness and, eventually, coma and even death.[[26](#_ENREF_26), [27](#_ENREF_27)]

Human rabies immune globulin (HRIG): Very mild and transient local reactions at the injection site such as pain and inflammation and systemic reactions such as headache, low grade fever, gastrointestinal disturbances, joint pain, and very rarely anaphylactic reactions, have been reported after receiving HRIG.[[28](#_ENREF_28), [29](#_ENREF_29)]

Confidentiality: There is a slight risk of loss of confidentiality, but every effort will be made to keep research information confidential as described in section [9.4.](#_Confidentiality_and_Privacy)

Photographic images: Taking pictures of the face and body may be embarrassing to some people. These photographs may be used for teaching or education purposes, as well as for future research projects by the study team or other researchers. They may also be published in medical journals, without identifying the participant. We will attempt to preserve the anonymity of the participant as much as possible, while providing the information needed to support the research being published. Photographs will be stored securely, but there is a slight risk of loss of confidentiality.

### Known Potential Benefits

Control participants with negative serum measles IgM and IgG will be provided measles vaccination by the study team or through the national immunization program. All participants with suspected measles will be provided Vitamin A by the study team according to WHO guidelines.[[30](#_ENREF_30)] This treatment replenishes deficient vitamin A levels during measles that occur even in well-nourished children and can help prevent eye damage and blindness as well as reduce mortality.[[30](#_ENREF_30), [31](#_ENREF_31)]

All participants will receive PrEP with Verorab (inactivated rabies vaccine) as part of the study. The WHO rabies PrEP recommendation includes a 2-dose option.[[32](#_ENREF_32)] Because the Sanofi Verorab rabies vaccine package leaflet [[33](#_ENREF_33)] has not yet been updated to include the 2-dose PrEP option and 3 doses is the current approved regimen in each of the proposed study countries, a 3-dose PrEP regimen will be used.

Rabies is estimated to cause 59,000 human deaths annually, and this number is thought to be a gross underestimate of the true burden of this disease. The highest mortality is in Africa with an estimated 21,476 deaths due to dog-mediated rabies each year.[[34](#_ENREF_34), [35](#_ENREF_35)] Because rabies is endemic in the regions where the study will be conducted, provision of rabies PrEP is a benefit to study participation. In the event there is a potential rabies exposure that occurs during the study, participants will be provided with post-exposure prophylaxis (PEP) with HRIG and rabies vaccine.


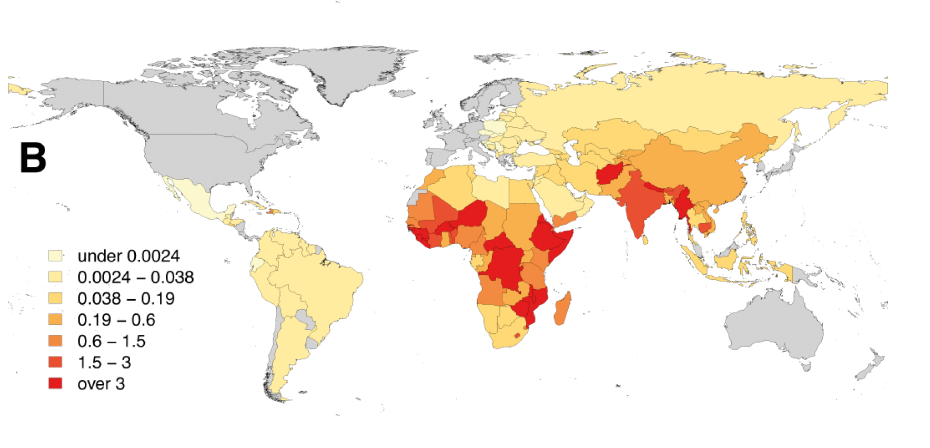


Death rates per capita (per 100 000 population) for dog-mediated rabies. Source :

<https://www.who.int/teams/control-of-neglected-tropical-diseases/rabies/epidemiology-and-burden>

### Assessment of Potential Risks and Benefits

Immune amnesia following MeV infection has the potential to negatively impact health, including an increased risk of serious endemic infections in West Africa, which poses a substantial threat to public health in this region and may disproportionally affect children. The information obtained in this study will contribute to a better understanding of these effects. Study participants will be exposed to minimal risk from biospecimen collection. The risk of vaccination with rabies vaccine is small and therefore outweighed by the potential benefits should a subsequent rabies virus exposure occur, as the vaccination is potentially lifesaving and rabies vaccines and HRIG PEP are often not locally affordable or available.[[36](#_ENREF_36)]

# STUDY OBJECTIVES

The objectives for this study are:

| **OBJECTIVES** | **ENDPOINTS** | **JUSTIFICATION FOR ENDPOINTS** |
| --- | --- | --- |
| **Primary** | | |
| To determine if MeV infection induces a loss of pre-existing immunity (immune amnesia) to endemic pathogens at Week 13 after baseline in children in select African regions. | Mean change in a panel of antibody levels over 13 weeks as measured by multiplex serological methods (e.g. PhIP-Seq/VirScan or SeroChip) and targeted ELISAs for confirmation. | These methods detect antibodies against multiple diverse pathogens and epitopes and assess both neutralizing and non-neutralizing antibodies. |
| To determine the effect of MeV infection on immune response to a controlled immune stimulus (rabies vaccination) at early and late timepoints post-infection. | Proportion of subjects with rabies virus neutralizing antibodies (RVNA) titer ≥ 0.5 International Units per milliliter (IU/mL) as measured by rapid fluorescent focus inhibition test 14 days after the last PrEP regimen vaccination. | These tests will measure the immune response and functionality of antibodies raised against the rabies vaccine. |
|  | Proportion of subjects with an RVNA titer ≥ lower limit of quantification 5-6 weeks after the first rabies vaccine dose. |  |
|  | Geometric mean RVNA titer 5-6 weeks after the first rabies vaccine dose. |  |
| **Secondary** | | |
| To determine if there is an increase in healthcare system encounters in the year following enrollment in children with recent MeV infection compared to those without recent MeV infection. | Mean number of non-study sick visit healthcare system encounters during the 1-year follow-up. | Visits to the healthcare system for sick visits in the year following enrollment in children with recent MeV infection will help determine the clinical impact of the immune amnesia in an objective manner. |
| To determine if MeV infection induces a loss of pre-existing immunity (immune amnesia) to endemic pathogens at Week 52 after baseline in children in select African regions. | Mean change in a panel of antibody levels over 52 weeks as measured by multiplex serological methods (e.g., PhIP-Seq/VirScan or SeroChip) and targeted ELISAs for confirmation. | These methods detect antibodies against multiple diverse pathogens and epitopes. These represent the spectrum of both neutralizing and non-neutralizing antibodies. |
| **Exploratory** | | |
| To assess the appearance and continued production of B and T cells, especially ASCs from PBMCs in circulation following MeV infection in children 1-15 years old in a subset of participants, depending on availability of collected blood samples. | Mean change in cell number and functionality from baseline to 14 days. | Assessing the number and functionality of the B and T cells including ASCs will give insight into the impact of MeV on B cell development and humoral immunity. |
| To attempt to identify the etiology of illness in children who present with signs and symptoms consistent with measles and are screened but not enrolled due to negative measles PCR and IgM. | Unbiased next generation sequencing for pathogen discovery directly from the oropharyngeal (OP)/nasopharyngeal (NP) swab. | This may identify other pathogens that may mimic the clinical presentation of MeV in children who have symptoms but test negative. |
| To identify the genotypes of MeV collected in the study. | Next generation sequencing or targeted Sanger sequencing directly from the OP/NP. | This will allow identification of different genotypes of MeV in the countries enrolling participants. |
| To characterize the phenotype of the rash and/or Koplik spots in children who present with signs and symptoms consistent with measles who test positive for measles (PCR and/or IgM positive) and who test negative for measles (PCR and IgM negative). | Differences in rash patterns/presentation in children who test positive versus those who test negative for measles based on analysis of photographs. | This will allow for a comparison of rash and/or Koplik spot presentation in potential cases that end up testing positive or negative for measles in the laboratory. This will complement the exploratory objective of identifying the etiology via unbiased next general sequencing in the participants who test negative for measles. In addition, we can contribute images of measles rash on dark skinned children and Koplik spots to the medical literature. |

# STUDY DESIGN

## Overall Design

This is a prospective, observational, longitudinal study to be conducted in select African regions to investigate the effects of MeV infection on pre-existing immunity, immune response to a known controlled immune stimulus (rabies vaccine), and susceptibility to infection to prevalent pathogens. Although children will receive the rabies vaccine as part of this study, we consider this an observational study rather than an interventional study. An interventional study tests an outcome of that intervention, e.g., efficacy or safety. The rabies vaccine is not used as a research intervention in this sense, i.e., we are not testing the efficacy or safety of the rabies vaccine, but rather using the vaccine as an immune stimulus to understand the effect of measles immune amnesia over time. In other words, the rabies vaccine is not the object of this investigation, but merely a means to allow assessment of the research objectives. Other vaccines could be used for this purpose, but rabies vaccine is convenient because there will not be pre-existing immunity to confound the results. The study is expected to take place in a subset of the NIAID Division of Clinical Research (DCR) Special Projects sites and any additional site(s) that may be identified. Specific study sites within each country will be in areas experiencing measles outbreaks.

We will enroll approximately 256 children aged 1-15 years into study Group 1 (acute MeV infection) or Group 2 (no acute measles infection). See section 5.1 for specific group criteria. In each group, approximately half of the enrolled participants will be ages 1-5 years and approximately half will be ages 6-15 years. Group 2 will be matched to Group 1 on gender and catchment area of study implementation.

Participants will undergo blood collection for screening and research testing at Day 0, between Days 7 and10 (optional, if Day 0 measles PCR and IgM are negative or equivocal in a child that presents with signs and symptoms consistent with measles), Day 14, Week 13, and Week 52. An upper respiratory specimen for MeV PCR will be collected at Day 0. Urine will be collected from females of reproductive potential to check for pregnancy at study visits with rabies vaccination or research blood collection. Hemoglobin levels will be checked at each study visit prior to research blood collection. Research samples will be tested for humoral and cellular immune responses to rabies vaccination (used as a controlled immune system stimulus, see section 8) and other research testing as described below (section 7.3). In addition, targeted sequencing will be performed on the measles virus to identify specific genotype. Unbiased sequencing will be used to try to identify the etiology in participants who have signs and symptoms consistent with measles but who test negative for measles by PCR and IgM. Optional photographs will be taken of the rash and/or Koplik spots at Day 0 to characterize the phenotype of the participants who test positive or negative for measles. We will also collect information on healthcare system encounters throughout the study period to help determine the clinical impact of immune amnesia after measles infection.

Participants with acute measles at screening/enrollment will be provided vitamin A treatment if they have not previously received it as part of their treatment. The treatment will follow WHO guidelines: 2 doses of 200,000 IU to be taken orally, 24 hours apart. The first dose will be given at the screening/enrollment visit, and the second dose will be given to the parent/guardian to be given the following day. A participant who has a rabies exposure during study participation will be provided rabies PEP with HRIG and rabies vaccine according to standard practice and the approved Verorab dosing schedule and depending on PrEP status. Any participant who receives rabies PEP prior to the timepoint that they were scheduled (randomized) to receive rabies PrEP under this study will continue in the study for follow-up visits but not receive PrEP according to their assigned randomization schedule. Data from these participants will be analyzed as described in Section [8.3](#_Population_for_Analysis).

Each site will have site-specific processes described in the manual of procedures (MOP) for activities, including but not limited to recruitment of cases and controls, informed consent and assent processes, laboratory processes, instructions for study procedures, and processes for follow-up of those with suspected MeV infection, HIV counseling and testing, and pregnancy testing.

## Study Sites

Details regarding local investigators, study sites, and enrollment targets for each site are outlined in the site-specific appendix (SSA) for each country participating in the study.

## Concomitant Therapy

For this protocol, a prescription medication is defined as a medication that can be prescribed only by a properly authorized/licensed clinician. Medications to be reported in the Case Report Form (CRF) are concomitant prescription medications, over-the-counter medications, and supplements.

# STUDY POPULATION

## Inclusion Criteria

To be eligible to participate in this study, an individual must meet all of the following criteria:

1. Aged 1 to 15 years.
2. Ability of the participant’s legal or culturally acceptable representative to provide informed consent.
3. Ability to give assent, as appropriate.
4. Stated willingness of parent/guardian and participant as appropriate, to comply with all study procedures.
5. Willingness to receive rabies vaccine.
6. Meet the criteria for assignment to Group 1 or Group 2, as follows:
   1. Group 1, cases (acute MeV infection):

- Clinical signs and symptoms suggestive of acute MeV infection (Koplik spots or skin rash) AND
- Laboratory confirmed measles:
  - - - - Upper respiratory specimen (swab) PCR for measles positive.

OR

- - - - - Serum IgM for measles positive.
  1. Group 2, controls (no acute MeV infection):
- No clinical signs and symptoms suggestive of acute MeV infection (Koplik spots or skin rash) AND
  - - - Upper respiratory specimen (swab) PCR negative for MeV AND
      - Serum measles IgM negative AND
      - Serum measles IgG positive and previously vaccinated for measles (2^nd^ dose will be offered if appropriate). If serum measles IgG is negative, participant must be willing to be vaccinated regardless of prior measles vaccine history to meet this criterion.

## Exclusion Criteria

An individual who meets any of the following criteria will be excluded from participation in this study:

1. HIV infection or any other immunosuppressive condition or medications.
2. Pregnant or lactating.
3. History of prior measles or immunologic evidence of prior measles in the absence of prior measles vaccination.
4. Severe anemia, defined as hemoglobin less than 8 g/dL.
5. Any acute or chronic condition which, in the opinion of the investigator, constitutes a contraindication to participation in this study.

**Co-enrollment guidelines:** Co-enrollment in other trials is restricted, other than enrollment on observational studies that do not exceed blood collection volume limits. Study staff should be notified of current and planned co-enrollment on any other protocol as it may require the approval of the principal investigator (PI).

## Inclusion of Vulnerable Participants

Children: We will only enroll children in this research protocol as this is the population most affected by measles and susceptible to the immune amnesia, which is the object of investigation in this study.

## Inclusion of Pregnant Women, Fetuses, or Neonates

Most of the females in our population will be below the age of menarche. Because pregnancy induces immunological changes that can confound the analysis of our data, pregnant persons will not be enrolled. Females who become pregnant during the study will be withdrawn from the study and no further study procedures will be done. Appropriate rabies vaccination PEP will be offered in the event of rabies exposure. Children less than of 1 year old will also not be included because they may retain maternal passive measles immunity for up to 1 year after birth.

## Screen Failures

Screen failures are defined as potential participants who consent to participate but do not meet inclusion/exclusion criteria for enrollment and are not subsequently enrolled in the study.

## Randomization

## Strategies for Recruitment and Retention

Participants will be recruited from the local population in each participating site’s catchment area. Site-specific processes for data mana will be provided in the SSA documents.

### Costs

There will be no costs associated with participation in this study.

### Compensation

Participants and the parent/guardian will be compensated for their time, transportation, and inconvenience based on locally acceptable and defined criteria at each site, as outlined in the SSA documents.

# PARTICIPANT DISCONTINUATION/WITHDRAWAL

## Participant Discontinuation/Withdrawal from the Study

Participants/parents/guardians are free to withdraw from participation in the study at any time.

An investigator may discontinue or withdraw a participant from the study for the following reasons:

- Significant non-compliance.
- If the participant meets an exclusion criterion (either newly developed or not previously recognized) that precludes further study participation, for example, pregnancy that occurs after enrollment.

The reason for participant discontinuation or withdrawal from the study will be recorded on the CRF.

## Lost to Follow-up

A participant will be considered lost to follow-up if he or she fails to return for follow-up visits according to criteria detailed in the MOP.

The following actions must be taken if a participant fails to return to the clinic for a required study visit:

- The site will attempt to contact the participant/parent/guardian to reschedule the missed visit, provide counsel on the importance of maintaining the assigned visit schedule, and ascertain if the participant or guardian/parent wishes to continue in the study. The participant may continue with the next scheduled visit, and the missed visit will not be considered a protocol deviation.
- These contact attempts should be documented in the participant’s study file.
- Should the participant continue to be unreachable until the time of the final study visit, he or she will be considered to be lost to follow-up.

# STUDY ASSESSMENTS AND PROCEDURES

The following study assessments and procedures will be performed according to the schedule provided in Table 1, Section 1.3.

## Screening Procedures

### Activities Performed Prior to Obtaining Informed Consent

Minimal risk activities that may be performed before the subject has signed a consent include the following:

- In-person, or telephone communications with prospective subjects or their parent/guardian.
- Review of existing medical records.
- Review existing radiographic images, if available.
- Review of existing photographs or videos, if available.
- Review of existing pathology specimens/reports from a specimen obtained for diagnostic purposes, if available.

### Activities Performed After a Consent for Screening Has Been Signed

The following screening procedures will be performed only after an informed consent has been signed by the participant/parent/guardian:

- Demographics review.
- Medical history collection.
- Physical exam, including vital signs (all participants) and mid upper arm circumference (MUAC; participants 12 to 59 months old).
- Concomitant medication.
- Vaccination history collection.
- Upper respiratory specimen (OP/NP swab) for measles PCR.
- Hemoglobin level.
- HIV testing.
- Urine pregnancy test for females of childbearing potential.
- Measles IgM/IgG serology.
- Optional photography.

All tests and procedures required to determine eligibility must be performed prior to enrollment; however, the Day 0 research blood sample and optional photography may be obtained once hemoglobin and pregnancy test (as applicable) results are confirmed, prior to confirmation of remaining screening test results. HIV testing will be performed with appropriate counseling per national policies. HIV testing for children younger than 24 months will be performed via PCR and repeated with Rapid Diagnostic Test (RDT) at Visit 11. HIV RDT will be performed at screening for participants 24 months and older and will not be repeated at Visit 11. Once all screening results are available, the participant will be notified of their eligibility status with a telephone call prior to the Day 14 study visit, if possible, or at the Day 14 study visit. If notified by phone, eligible participants will be scheduled for a Day 14 study visit and ineligible participants will be invited to site to discuss test results and referred for measles vaccination if both serum IgM and IgG antibody tests are negative.

## Description of Study Procedures

Measles vaccination: Participants who enroll as controls and are found to have a negative measles IgM and IgG serology will be provided measles vaccination by the study team or referred to receive the measles vaccine through the national immunization program to ensure these participants are protected against measles and do not develop a natural MeV infection during the study period. Control participants who have evidence of immunity to measles at screening will not be vaccinated.

Pregnancy test: Urine pregnancy testing will be performed.

Medical history, vaccination history, and concomitant medications: Medical history, vaccination history, and concomitant medications will be reviewed at screening and updated at indicated study visits (see Table 1).

Rabies vaccination: A 3-dose series of Sanofi Verorab (inactivated rabies vaccine) will be given according to the manufacturer’s standard dosing instructions. Participants will be randomized to receive the series at an early (Week 8) or late (Week 47) timepoint (see Table 1 for specific dosing timepoints).

Physical examination: Physical exam will include measurements of height, weight, vital signs (temperature, heart rate, and respiratory rate) for all participants and anthropomorphic measurement (MUAC) in children 12 to 59 months as indicated in Table 1, Schedule of Activities. At Visit 0, the physical exam will include an assessment for clinical signs and symptoms suggestive of acute MeV infection (e.g., skin rash).

Upper respiratory specimen: OP/NP swabs will be collected for PCR testing to diagnose measles and for research evaluations (see Section 7.3).

Blood draw: Blood samples will be drawn via finger/heel stick and/or venipuncture according to standard site procedures for laboratory evaluations and storage. The amount of blood drawn for research purposes at each visit (no more than 5 mL) will be within the limits allowed for research participants in each country and will be specified in the sites’ SSAs. If additional research samples or testing will be performed at certain sites, the tests, timing of sample collection, type of sample, and amount of blood, if applicable, must be described in the SSA and MOP.

Assessment of adverse events (AEs): AEs and serious adverse events (SAEs) that are related to the research procedures described in this protocol will be recorded, except for Grade 1 or 2 AEs that are expected. AEs and SAEs that in the investigators judgment are not at least potentially related to research procedures, for example those that are due to the natural course of the disease, will not be recorded as AEs/SAEs in the research database. All SAEs will be reported to local authorities and ECs per local reporting requirements.

Healthcare utilization: The study team will collect information on healthcare encounters, to the extent possible, during the year-long follow-up to determine if there is a difference in the number of encounters by study group.

Photography (optional): The study team may take photographs of the skin rash and/or Koplik spots during the screening/enrollment visit to document the signs of measles and non-measles. The photographs may include the face and eyes but will not include the whole face. All photographs will be stored securely.

## Biospecimen Evaluations and Correlative Studies for Research

Biospecimens and data collected under this protocol will be used to study immune amnesia following MeV infection and the immune response to a controlled immune stimulus (rabies vaccine). Of the 5 mL of blood obtained through venipuncture, plasma will be saved to measure rabies virus antibody responses and global antibody responses for immune amnesia. Cells will be saved to address the aims associated with B and T cells including ASCs. Stored specimens may be used to address research questions related to the main study objectives, for example, additional measurements of antibody responses by various assays, measurements of biomarkers that may influence vaccine responsiveness, additional analysis of ASCs, analysis of B and T cells, and other assays studying PBMC functionality. PBMCs will be collected in a subset of participants. The upper respiratory specimens (swabs) will be used for measles virus sequencing. Plasma or upper respiratory specimens (swabs) may be used for unbiased sequencing for viruses or bacteria. All sequencing and analyses will target pathogen (viral, bacterial, etc.) genetic material only, and no human genome data will be generated. All collected research specimens will be stored for later assessment. The specimen collection procedures will be specified in the Laboratory MOP.

## Management/Return of Results

Any clinically relevant test results such as a pregnancy, measles infection, measles IgG, measles IgM, measles PCR, HIV infection, or anemia will be shared with the participant. If the rabies neutralizing results are below the cutoff for protection, attempts to share the results with the participant may be made. Results from research laboratory assays (such as immunologic responses) will not be returned to participants/parents/guardians.

## Adverse Events

### Definitions

AE: Any untoward medical occurrence in a human subject, including any abnormal sign (for example, abnormal physical exam or laboratory finding), symptom, or disease, temporally associated with the subject’s participation in research, whether or not considered related to the subject’s participation in the research.

SAE: An SAE is any AE that:

- Results in death;
- Is life-threatening (places the subject at immediate risk of death from the event as it occurred);
- Results in inpatient hospitalization or prolongation of existing hospitalization;
- Results in a persistent or significant disability/incapacity;
- Results in a congenital anomaly/birth defect; OR
- Based upon appropriate medical judgment, may jeopardize the subject’s health, and may require medical or surgical intervention to prevent one of the other outcomes listed above in this definition (examples of such events include allergic bronchospasm requiring intensive treatment in the emergency room or at home, blood dyscrasias or convulsions that do not result in inpatient hospitalization, or the development of drug dependency or drug abuse).

Unanticipated Problem (UP): Any incident, experience, or outcome that meets all of the following criteria:

- Unexpected in terms of nature, severity, or frequency given (a) the research procedures that are described in the protocol-related documents, such as the IRB/EC-approved research protocol and informed consent document; and (b) the characteristics of the participant population being studied; and
- Related or possibly related to participation in the research (“possibly related” means there is a reasonable possibility that the incident, experience, or outcome may have been caused by the procedures involved in the research); and
- Suggests that the research places participants or others (which many include research staff, family members or other individuals not directly participating in the research) at a greater risk of harm (including physical, psychological, economic, or social harm) than was previously known or expected.

## Classification of an Adverse Event

### Severity of Event

AEs will be graded according to the “Division of AIDS Table for Grading the Severity of Adult and Pediatric Adverse Events” Corrected Version 2.1, July 2017 (<https://rsc.niaid.nih.gov/sites/default/files/daidsgradingcorrectedv21.pdf>).

### Relationship to Study Procedures

All AEs will have their relationship to study procedures assessed by an appropriately trained clinician based on temporal relationship and his/her clinical judgment. The degree of certainty about causality will be graded using the categories below.

**Related** – The AE is known to occur with the study procedures, there is a reasonable possibility that the study procedures caused the AE, or there is a temporal relationship between the study procedures and the event. Reasonable possibility means that there is evidence to suggest a causal relationship between the study procedures and the AE.

**Not Related** – There is not a reasonable possibility that the study procedures caused the event, there is no temporal relationship between the study procedures and event onset, or an alternate etiology has been established.

#### Expectedness

A clinician with appropriate expertise in infectious diseases and vaccination will be responsible for determining whether an adverse event (AE) is expected or unexpected. An AE will be considered unexpected if the nature, severity, or frequency of the event is not consistent with the risk information previously described for the study procedures. Expected AEs can be found in Section 2.3.1.

### Type and Duration of the Follow-up of Participants After AEs

AEs related to study procedures will be followed through resolution or until the local investigator judges that the event has stabilized, and no additional follow-up is required.

## Reporting

SAEs and UPs will be reported to Sanofi Pasteur according to the terms of the Clinical Trials Agreement.

Safety events will be tracked and submitted to the local IRB/EC according to each site’s/country’s requirements. These are described in the SSAs.

# STATISTICAL CONSIDERATIONS

This is a prospective, observational, longitudinal study. Children with acute measles and a control group without measles will be enrolled and followed for 1 year to examine the impact of MeV on the immune system. The impact will be measured using laboratory techniques that can assess the changes in total antibody diversity to multiple pathogens.

A controlled immune stimulus consisting of the rabies vaccine will be given to all participants with half receiving vaccines shortly after enrollment (at Week 8; “early” group) and the other half receiving them at a later timepoint (Week 47; “late” group). Antibodies to the rabies vaccine will be measured 5 weeks after the first rabies vaccine. This will provide information on the development of the immune response following MeV when the immune system is challenged with a controlled stimulus. The administration of early and late rabies vaccine will be randomly assigned and stratified by measles, by country group, and by age group.

## Statistical Hypothesis

1. There is a difference in antibody level changes from baseline to Week 13 between the acute MeV group and the control group.
2. There is a difference in rabies antibody titer between the acute MeV infection and the control groups at early and late timepoints.

## Sample Size Determination

The overall sample size will be 256 participants. We will accrue 128 acute measles participants (with half of these in age group 1-5 years and half in age group 6-15 years) and 128 control participants (with half in each age group).

Randomization to early and late rabies vaccine will be stratified by country, group, and age.

Power for comparison of rabies titers

The sample size calculation is based on detecting a difference in the rabies antibody geometric mean titer between the MeV and control groups. The rabies vaccine will be given to half of the participants at Week 8 and half at Week 47 post enrollment, and the rabies antibody titer will be measured 5-6 weeks after receipt of the first vaccine dose (Weeks 13 and 52, respectively). There will be a random assignment for receipt of rabies vaccination to the two timepoints with equal numbers in the acute measles young and old groups and control young and old groups. In this analysis only one measurement of rabies antibody titer per participant will be included and it will be the measurement 5 weeks after receiving the rabies vaccine.

The total study sample size will be divided into 8 cohorts stratified by measles status, age group, and rabies vaccine timepoint as outlined in Figure 1. The standardized difference that can be detected with 90% power using a 0.05 level two-sided test for different sample sizes by specific cohort are shown in [Table 2.](#_Table_2:_Standardized)

Table 2: Standardized detectable difference with 90% power using a 0.05 level two-sided test for different sample sizes.

| Sample size per specific cohort (total study sample size) | Comparison of MeV to control at either the early time point or late time point  (assume std=1) | Interaction between time and group.  Example: comparison of difference between early MeV and control vs late MeV and control  (assume std=(1^2^+1^2^)^.5^=1.4) |
| --- | --- | --- |
| 20 (160) | 1.05 | 1.47 |
| 30 (240) | 0.85 | 1.19 |
| 40 (320) | 0.73 | 1.02 |

The chosen sample size of 256 accounts for a 6.5% loss to follow up.

Power for multiplex serological methods for global antibody analysis (e.g. virscan):

We also present sample size calculations to show detectable change over time between groups in the panel of antibody measurements. This analysis will compare change in the panel of measurements from baseline to the 3-month blood draw between groups. The panel will contain numerous measurements, so we will use a Bonferroni correction to control the study-wide error rate at p=0.05. To be conservative, we will assume 1000 comparisons and plan to maintain the alpha level at a two-sided 0.05 rate (thus the power calculations will be performed with alpha=0.025/1000). We will perform the analysis separately for children aged 1-5 years and those aged 6-15 years. Table 3 gives the detectable between-group standardized difference (difference/std) with 90% power for different samples sizes with the Bonferroni correction for multiple comparisons. Here we combine the early and late rabies randomization groups since the rabies vaccination should not affect the comparison of other antibodies.

Table 3. Detectable standardized differences with 90% power.

The sample size is the number needed in an arm (measles or control), age group (young vs old (multiply by 4 for total sample size)

| Sample size per arm (total study sample size) | Standardized detectable difference |
| --- | --- |
| 40 (160) | 1.26 |
| 60 (240) | 1.00 |
| 80 (320) | 0.86 |

Note: The detectable difference of 1.00 that can be seen with a sample size of 240 is well within what the Mina paper found and is a reasonable sample size for this study.[[12](#_ENREF_12)]

Power for medical visit endpoint.

A particularly important secondary endpoint is healthcare encounters. We have calculated the power to compare health care encounters between the MeV and control groups for the selected sample size. One method to summarize healthcare encounters is to look at the proportion of participants in each group that had at least one medical visit. Using this measure, there would be 90% power to detect a difference in proportions of 0.5 versus 0.7 (control group versus MeV group, respectively) using a test of proportions. Note a proportion of 0.5 for the control group was used since this proportion gives the largest variance. Any larger or smaller proportions will allow smaller difference in proportions to be detected with 90% power. In the analysis, the Poisson test may be used which would be more powerful if participants have multiple healthcare encounters.

## Feasibility Assessment

The feasibility of the study will be assessed by NIAID the sponsor within 12 months of enrolling the initial participant. If less than 90% of the study sample (with day 14 confirmation of eligibility) has been enrolled, the study sponsor will consider terminating the study enrollment and/or follow-up.

## Population for Analysis

All participants enrolled in the study will be analyzed. Any control participants who later becomes infected with measles or participants from either group that become pregnant will be censored at the time of infection or pregnancy.

If a participant who was enrolled as a case is later diagnosed with measles during the study, there is no change with regards to how their data will be analyzed or with regards to other study procedures. However, as a measles infection should confer lifelong immunity, this scenario is expected to be extremely unlikely.

A participant who has received rabies PEP during the study prior to the timepoint at which they were scheduled (randomized) to receive rabies vaccination will be excluded from the primary analysis of the rabies vaccine but included in the immune amnesia analysis.

Participants who have received their first dose of rabies PrEP as scheduled (randomized) will be analyzed as planned.

Participants who do not receive a full course of rabies vaccine will be included in all analyses. A sensitivity analysis will be performed where these participants are excluded.

## Statistical Analyses

### General Approach

Demographic variables will be summarized overall by group and by group and country. Binary endpoints will be reported as proportions with 95% Wilson score confidence intervals (CIs) or logistic regression model coefficients and CIs. Continuous endpoints will be summarized with means and standard deviations and/or geometric means and CIs based on the t-distribution or using regression models. Participants with missing data may be dropped from the analysis.

### Analysis of Primary Endpoints

The analysis of the rabies antibody will be performed using a 0.05 level two-sided test as described in the sample size determination (section 8.2). 95% CIs around the parameter estimates will be provided. The panel of antibodies will be analyzed using t-tests and controlling the 2-sided 0.05 error rate via a Westfall Young approach as this will be more powerful than a Bonferroni correction.

### Analysis of Secondary Endpoints

The proportion of participants hospitalized between groups will be reported, exact conditional tests will be performed, and 95% Wilson score CIs will be calculated. A Poisson analysis will be used to compare the number of encounters with medical providers between groups. The average number of encounters per group per person will be calculated and compared. Since each person will have 1 year of follow-up, this would be appropriate. If participants drop out, we will adjust for time in study for each person.

# REGULATORY AND OPERATIONAL CONSIDERATIONS

## Informed Consent Process

### Consent/Assent Procedures and Documentation

Informed consent is a process where information is presented to enable persons to voluntarily decide whether or not to participate as a research participant. It is an ongoing conversation between the human research participant/parent/guardian and the researchers which begins before consent is given and continues until the end of the participant’s involvement in the research. Discussions about the research will provide essential information about the study and include purpose, duration, experimental procedures, alternatives, risks, and benefits. Coercion and undue influence will be minimized by informing participants and their parents/guardians that the decision to join the study will not affect any current medical care. Participants/parents/guardians will be given as much time as they need to read the consent/assent form, or have it read to them if unable to read and ask questions of the investigators. Participants/parents/guardians will also be given time to discuss their participation with family members, friends, and other healthcare providers.

Participant assent and parental/guardian permission will be obtained according to local standards and country-specific requirements and will be described in the SSA. The participant/parent/guardian will sign the informed consent document (in a language understood by the participant) and the eligible participants will sign the assent document (as applicable) prior to any procedures being done specifically for the study. Details of informed consent and assent procedures will be described in the MOP. In settings where assent will be conducted, minors who decline participation in the study after reviewing the assent materials will not be enrolled even if their parent(s) or legal guardian consent to their participation.

Informed consent/assent will be obtained in person by a study team member authorized to obtain consent. The privacy of the participant will be maintained. The consenting investigator and participant/parent/guardian will be located in a private area (e.g., clinic consult room).

Informed consent of minors will be provided by one of the parents or the child’s legal guardian, except in the case of emancipated minors (such as a minor who is married or a parent), who may provide their own consent without parental/guardian permission.

A copy of the informed consent/assent documents will be given to the participants for their records. The consenting investigator will document the signing of the consent/assent forms in the participant’s study record. The investigator will confirm that written legally effective consent has been obtained prior to initiating any study interventions.

The rights and welfare of the participants will be protected by emphasizing to them that the quality of their medical care will not be adversely affected if they decline to participate in this study. The participants may withdraw consent at any time throughout the course of the trial.

## Study Discontinuation and Closure

This study may be temporarily suspended or prematurely terminated if there is sufficient reasonable cause. Suspension or termination may only apply to a single site or country and not affect conduct of the study in other sites or countries. Written notification, documenting the reason for study suspension or termination, will be provided by the suspending or terminating party to study participants, investigators, and the IRBs/ECs. Study participants/parents/guardians will be contacted, as applicable, and be informed of changes to the study visit schedule.

Circumstances that may warrant termination or suspension include, but are not limited to:

- Determination of unexpected, significant, or unacceptable risk to participants.
- Insufficient compliance to protocol requirements.
- Data that are not sufficiently complete and/or evaluable.

The study may resume once concerns about safety, protocol compliance, and data quality are addressed and satisfy the IRBs/ECs and sponsor, as applicable.

## Future Use of Stored Specimens and Data

**Intended Use:** Coded data and specimens will be stored indefinitely for future research related to measles and other infectious diseases after the study is complete.

**Storage:** All of the stored research samples and data are labeled by a code that only the local investigators can link to the participant. The key to the participant codes will be maintained securely at the study sites. NIH investigators will not have access to the key code or any identifiers. Study samples will be stored for analysis in repositories in each participating country. While every effort will be made to ensure analyses occur in-country, some export of samples to central laboratories in the US or other region may be necessary. In each country, sample storage will be in a secure facility with access limited to research staff according to the site’s MOP. CRFs will be retained in secure facilities at the study sites. Data will be kept in password-protected computers. Only investigators or their designees will have access to the samples and data. If sites are planning to collect additional samples and tests, these must be clearly described in the informed consent form, SSA and the MOP, with special attention to storage and testing details and receive appropriate local IRB/EC review and approval.

**Disposition:** In the future, other investigators may wish to use these samples and/or data for research purposes. If the planned research falls within the category of “human subjects research” on the part of the researchers, local IRB/EC review and approval will be obtained. This includes the researchers sending out coded and linked samples or data and getting results that they can link back to their subjects. Countries will include specific policy language related to sample sharing and data usage in their SSA or other site-specific documents.

**Loss or Destruction:** Any loss or unanticipated destruction of samples (for example, due to freezer malfunction) or data (for example, misplacing a printout of data with identifiers) that meets the definition of a reportable event will be reported to the local IRB/EC.

Additionally, participants may decide at any point not to have their samples stored. In this case, the PI will destroy all known remaining samples and report what was done to both the participant and to the local IRB/EC. This decision will not affect the individual’s participation in this protocol.

## Confidentiality and Privacy

All records will be kept confidential to the extent provided by federal, state, and local law. The study monitors and other authorized individuals may inspect all documents and records required to be maintained by the investigator, including but not limited to, medical records. Records will be kept locked, and data will be coded. Any personally identifiable information maintained for this study will be kept on restricted-access computers and networks. Personally identifiable information will only be shared with individuals authorized to receive it under this protocol. Individuals not authorized to receive personally identifiable information will be provided with coded information only, as needed. Clinical information will not be released without written permission of the participant, except as necessary for monitoring by the IRBs/ECs or representatives of NIAID.

## Study Monitoring

### Data and Safety Monitoring

The data gathered during this study will be monitored by the site PIs for safety and compliance with protocol-specified requirements. Each site team will establish a plan to regularly review consents, completion of CRFs, and safety reporting.

### Clinical Monitoring

According to the ICH E6(R2) GCP guidelines, section 5.18, and FDA 21 CFR 312.50, clinical protocols are required to be adequately monitored by the study sponsor. This study monitoring will be conducted according to the “NIAID Intramural Clinical Monitoring Guidelines.” Monitors under contract to the NIAID/Office of Clinical Research Policy and Regulatory Operations (OCRPRO) will visit the clinical research site in person or virtually to monitor aspects of the study in accordance with the appropriate regulations and the approved protocol. The objectives of a monitoring visit will be: 1) to verify the existence of signed informed consent documents and documentation of the consent process for each monitored participant; 2) to verify the prompt and accurate recording of all monitored data points in the study database and prompt reporting of all SAEs; 3) to compare abstracted information entered into the study database with individual participants’ records and source documents (participants’ charts, laboratory analyses and test results, physicians’ progress notes, nurses’ notes, and any other relevant original participant information); and 4) to help ensure investigators are in compliance with the protocol. The monitors also will inspect the clinical site regulatory files to ensure that regulatory requirements (Office for Human Research Protections) and applicable guidelines (ICH GCP) are being followed. During the monitoring visits, the investigator (and/or designee) and other study personnel will be available to discuss the study progress and monitoring visit.

The investigator (and/or designee) will make study documents (e.g., consent forms, database data abstracts) and pertinent hospital or clinical records readily available for inspection by the local IRB/EC, the site monitors, and the NIAID staff for confirmation of the study data.

A specific protocol monitoring plan will be discussed with the PI and study staff prior to enrollment. The plan will outline the frequency of monitoring visits based on such factors as study enrollment, data collection status, and regulatory obligations.

## Quality Assurance and Quality Control

To help ensure that NIH Office of Research Support and Compliance procedures and GCP are being carried out, a Clinical Trials Management designee within the OCRPRO Regulatory Compliance and Human Subjects Protection Program will conduct a study initiation visit before study enrollment begins. The purpose of this meeting is to review with the investigators and study team designees the roles and responsibilities concerning their commitment to adhere to the requirements of the protocol, especially in terms of NIH Office of Human Subjects Research Protections reporting requirements for reportable events. In addition, the quality management and data management plan for the study will be reviewed.

## Data Handling and Record Keeping

### Data Collection and Management Responsibilities

Study data will be maintained in a designated DFdiscover database and collected directly from participants/parents/guardians during study visits or will be abstracted from participants’ medical and laboratory records. Source documents include all recordings of observations or notations of clinical activities and all reports and records necessary to confirm the data abstracted for this study. Designated CRFs will also serve as source documents. Data entry onto paper and/or electronic CRFs and into the study database will be performed by authorized individuals. The investigator is responsible for assuring that the data collected are complete, accurate, and recorded in a timely manner. Study data, including cumulative subject accrual numbers, should be generated via the chosen data capture method and submitted to the IRB/EC as needed.

### Study Records Retention

The site investigators will be responsible for retaining all essential documents listed in the ICH GCP guideline. Study records will be maintained according to applicable NIH, sponsor, and local policies. No records will be destroyed without the written consent of the PI and sponsor, as applicable.

Should the investigator wish to assign the study records to another party and/or move them to another location, the investigator will provide written notification of such intent to OCRPRO/NIAID with the name of the person who will accept responsibility for the transferred records and/or their new location. Relocation of research records will not proceed without written permission from OCRPRO/NIAID.

## Protocol Deviations and Non-Compliance

It is the responsibility of the investigator to use continuous vigilance to identify and report deviations and/or non-compliance to the IRBs/ECs per local policy. All deviations must be addressed in study source documents and reported as specified in the protocol quality management plan and/or monitoring plan. The investigator is responsible for knowing and adhering to the reviewing IRB/EC requirements.

A protocol deviation is any change, divergence, or departure from the IRB/EC-approved research protocol.

- Major deviations: Deviations from the IRB/EC-approved protocol that have or may have the potential to negatively impact the rights, welfare, or safety of the subject or to substantially negatively impact the scientific integrity or validity of the study.
- Minor deviations: Deviations that do not have the potential to negatively impact the rights, safety, or welfare of subjects or others or the scientific integrity or validity of the study.

## Reporting to the NIAID Clinical Director

UPs, major protocol deviations, and deaths will be reported to the NIAID clinical director according to institutional timelines.

## Publication and Data Sharing Policy

### Human Data Sharing Plan

This study will be conducted in accordance with the following publication and data sharing policies and regulations:

NIH Public Access Policy, which ensures that the public has access to the published results of NIH-funded research. It requires scientists to submit final peer-reviewed journal manuscripts that arise from NIH funds to the digital archive PubMed Central upon acceptance for publication.

This study will comply with the NIH Data Management and Sharing Policy and Policy on the Dissemination of NIH-Funded Clinical Trial Information and the Clinical Trials Registration and Results Information Submission rule. As such, this study will be registered at ClinicalTrials.gov. In addition, every attempt will be made to publish results in peer-reviewed journals.

### Genomic Data Sharing Compliance

This study will comply with the NIH Genomic Data Sharing Policy, which applies to all NIH-funded research that generates large-scale non-human genomic data and for the use of these data for subsequent research. Pathogen sequence data will be deposited in GenBank. No human genomic sequencing will be done.

## Collaborative Agreements

### Agreement Type

Coded data will be shared with Sanofi Pasteur through a confidential Clinical Trials Agreement.

## Conflict of Interest Policy

The independence of this study from any actual or perceived influence is critical. Therefore, any actual conflict of interest of persons who have a role in the design, conduct, analysis, publication, or any aspect of this study will be disclosed and managed. Furthermore, persons who have a perceived conflict of interest will be required to have such conflicts managed in a way that is appropriate to their participation in the design and conduct of this study.

# ABBREVIATIONS

| AE | Adverse Event |
| --- | --- |
| ASC | Antibody Secreting Cells |
| CI | Confidence Interval |
| CRF | Case Report Form |
| DCR | Division of Clinical Research |
| EC | Ethics Committee |
| GCP | Good Clinical Practice |
| HIV | Human Immunodeficiency Virus |
| HRIG | Human Rabies Immune Globulin |
| ICH | International Council for Harmonisation of Technical Requirements for Pharmaceuticals for Human Use |
| Ig | Immunoglobulin |
| IRB | Institutional Review Board |
| MeV | Measles Virus |
| MOP | Manual of Procedures |
| MUAC | Mid-Upper Arm Circumference |
| NIAID | National Institute of Allergy and Infectious Disease |
| NIH | National Institutes of Health |
| NP | Nasopharyngeal |
| OCRPRO | Office of Clinical Research Policy and Regulatory Operations |
| OP | Oropharyngeal |
| PBMC | Peripheral Blood Mononuclear Cell |
| PCR | Polymerase Chain Reaction |
| PEP | Post Exposure Prophylaxis |
| PI | Principal Investigator |
| PrEP | Pre-Exposure Prophylaxis |
| RDT | Rapid Diagnostic Test |
| RVNA | Rabies Virus Neutralizing Antibodies |
| SAE | Serious Adverse Event |
| SSA | Site-Specific Appendix |
| UP | Unanticipated Problem |
| WHO | World Health Organization |

# REFERENCES

1. UNICEF. Guinea Country Office Humanitarian Situation Report No. 1. Available at: <https://www.unicef.org/media/124941/file/Guinea-Humanitarian-SitRep-January-June-2022.pdf>. Accessed August 26.

2. UNICEF. Mali Humanitarian Situation Report No. 2. Available at: <https://reliefweb.int/report/mali/unicef-mali-humanitarian-situation-report-no-2-30-april-2022>. Accessed August 26.

3. National Public Health Institute of Liberia. Liberia IDSR Epidemiology Bulletin, Epi-week 42 (October 17-23, 2022). **2022**; 17(42).

4. SeyedAlinaghi S, Karimi A, Mojdeganlou H, et al. Impact of COVID-19 pandemic on routine vaccination coverage of children and adolescents: A systematic review. Health Sci Rep **2022**; 5(2): e00516.

5. Shet A, Carr K, Danovaro-Holliday MC, et al. Impact of the SARS-CoV-2 pandemic on routine immunisation services: evidence of disruption and recovery from 170 countries and territories. Lancet Glob Health **2022**; 10(2): e186-e94.

6. Truelove SA, Moss WJ, Lessler J. Mitigating measles outbreaks in West Africa post-Ebola. Expert Rev Anti Infect Ther **2015**; 13(11): 1299-301.

7. Frenkel LD. The global burden of vaccine-preventable infectious diseases in children less than 5 years of age: Implications for COVID-19 vaccination. How can we do better? Allergy Asthma Proc **2021**; 42(5): 378-85.

8. Vittrup DM, Laursen ACL, Malon M, et al. Measles-mumps-rubella vaccine at 6 months of age, immunology, and childhood morbidity in a high-income setting: study protocol for a randomized controlled trial. Trials **2020**; 21(1): 1015.

9. WHO. Measles Fact Sheet. Available at: <https://www.who.int/en/news-room/fact-sheets/detail/measles>. Accessed August 26.

10. Griffin DE. Measles Vaccine. Viral Immunol **2018**; 31(2): 86-95.

11. Griffin DE. Measles immunity and immunosuppression. Curr Opin Virol **2021**; 46: 9-14.

12. Mina MJ, Kula T, Leng Y, et al. Measles virus infection diminishes preexisting antibodies that offer protection from other pathogens. Science **2019**; 366(6465): 599-606.

13. Petrova VN, Sawatsky B, Han AX, et al. Incomplete genetic reconstitution of B cell pools contributes to prolonged immunosuppression after measles. Sci Immunol **2019**; 4(41).

14. Aaby P, Bhuiya A, Nahar L, Knudsen K, de Francisco A, Strong M. The survival benefit of measles immunization may not be explained entirely by the prevention of measles disease: a community study from rural Bangladesh. Int J Epidemiol **2003**; 32(1): 106-16.

15. Aaby P, Samb B, Simondon F, Seck AM, Knudsen K, Whittle H. Non-specific beneficial effect of measles immunisation: analysis of mortality studies from developing countries. BMJ **1995**; 311(7003): 481-5.

16. Higgins JP, Soares-Weiser K, Lopez-Lopez JA, et al. Association of BCG, DTP, and measles containing vaccines with childhood mortality: systematic review. BMJ **2016**; 355: i5170.

17. Mina MJ. Measles, immune suppression and vaccination: direct and indirect nonspecific vaccine benefits. J Infect **2017**; 74 Suppl 1: S10-S7.

18. Sato R, Haraguchi M. Effect of measles prevalence and vaccination coverage on other disease burden: evidence of measles immune amnesia in 46 African countries. Hum Vaccin Immunother **2021**; 17(12): 5361-6.

19. Sorup S, Benn CS, Poulsen A, Krause TG, Aaby P, Ravn H. Live vaccine against measles, mumps, and rubella and the risk of hospital admissions for nontargeted infections. JAMA **2014**; 311(8): 826-35.

20. Behrens L, Cherry JD, Heininger U, Swiss Measles Immune Amnesia Study G. The Susceptibility to Other Infectious Diseases Following Measles During a Three Year Observation Period in Switzerland. Pediatr Infect Dis J **2020**; 39(6): 478-82.

21. Buhl D, Staudacher O, Santibanez S, et al. Specifically Increased Rate of Infections in Children Post Measles in a High Resource Setting. Front Pediatr **2022**; 10: 896086.

22. Gadroen K, Dodd CN, Masclee GMC, et al. Impact and longevity of measles-associated immune suppression: a matched cohort study using data from the THIN general practice database in the UK. BMJ Open **2018**; 8(11): e021465.

23. Le NTH, Ho NT, Grenfell B, Baker S, Geskus RB. Biphasic pattern in the effect of severe measles infection; the difference between additive and multiplicative scale. BMC Infect Dis **2021**; 21(1): 1249.

24. Laksono BM, de Vries RD, Verburgh RJ, et al. Studies into the mechanism of measles-associated immune suppression during a measles outbreak in the Netherlands. Nat Commun **2018**; 9(1): 4944.

25. McEldrew EP, Lopez MJ, Milstein H. Vitamin A. StatPearls. Treasure Island (FL), **2023**.

26. Vitamin A. LiverTox: Clinical and Research Information on Drug-Induced Liver Injury. Bethesda (MD), **2012**.

27. US National Institutes of Health Office of Dietary Supplements. Vitamin A and Carotenoids - Fact Sheet for Health Professionals. Available at: <https://ods.od.nih.gov/factsheets/VitaminA-HealthProfessional/>. Accessed May 3, 2023.

28. Medecins Sans Frontieres (Doctors Without Borders). MSF medical guidelines: Human Rabies Immunoglobulin (HRIG). Available at: <https://medicalguidelines.msf.org/en/viewport/EssDr/english/human-rabies-immunoglobulin-hrig-16688380.html#section-target-5>. Accessed May 4, 2023.

29. US Centers for Disease Control and Prevention. Rabies: Adverse Reaction. Available at: <https://www.cdc.gov/rabies/specific_groups/hcp/adverse_reaction.html>. Accessed May 4, 2023.

30. WHO. Newsroom, Fact Sheets: Measles. Available at: <https://www.who.int/news-room/fact-sheets/detail/measles>. Accessed May 4, 2023.

31. D'Souza RM, D'Souza R. Vitamin A for treating measles in children. Cochrane Database Syst Rev **2002**; (1): CD001479.

32. O'Brien KL, Nolan T, Rabies SWo. The WHO position on rabies immunization - 2018 updates. Vaccine **2019**; 37 Suppl 1(Suppl 1): A85-A7.

33. Sanofi Pasteur Ltd. Verorab Package Leaflet. Available at: <https://www.sanofi.com.eg/dam/jcr:7debf4ec-9323-4395-9f2f-fe9bc3589bf6/Verorab.pdf>. Accessed January 5.

34. WHO. WHO Expert Consultation on Rabies: WHO TRS No. 1012. Available at: <https://www.who.int/publications/i/item/WHO-TRS-1012>. Accessed 23 March 2023.

35. WHO. Control of Neglected Tropical Diseases: Rabies, Epidemiology and burden of disease: Global burden of dog-transmitted human rabies. Available at: <https://www.who.int/teams/control-of-neglected-tropical-diseases/rabies/epidemiology-and-burden>. Accessed 23 March 2023.

36. WHO Regional Office for Africa. WHO Africa: Health topics, Rabies. Available at: <https://www.afro.who.int/health-topics/rabies>. Accessed December 27.
