# Supplementary material for: Investigating immune amnesia after measles virus infection in two West African countries: A study protocol
Source: PLoS One. 2025 Jun 26;20(6):e0314828. doi: 10.1371/journal.pone.0314828 (PMC12200839; doi:10.1371/journal.pone.0314828)
Supplement: Attachment — Submitted filename: Review.docx [file pone.0314828.s003.docx]

**"Investigating immune amnesia after measles virus infection in two West African countries: A study protocol"**

This manuscript presents an important study that could significantly contribute to our understanding of immune amnesia associated with measles infection. Addressing the points mentioned above will enhance clarity, transparency, and rigor, ultimately supporting the validity of the research findings.

I'll be looking at clarity, structure, grammar and relevance of content. This article needs to be read by a native English speaker.

**Title:**

The title is informative and precise. However, it could benefit from simplification, for example:

- “Study of immune amnesia following measles virus infection in West Africa”.

**Abstract:**

What is the working hypothesis?

Line 16: “Investigation of Immune Amnesia Following Measles Infection in Select African Regions.”

Correction: Make sure this sentence aligns with the article title.

Line17-18: “a prospective, observational, longitudinal study being conducted in two West African countries, Guinea, and Mali.”

Correction: Delete the comma after “Guinea”.

**Material and methods**

Line 86**:** Although you mention enrolling 256 children, further justification of this sample size in terms of statistical power and expected effect sizes would strengthen the manuscript. Please explain the basis for this number and if any preliminary data guided this decision

Line 87: “Acute MeV is confirmed by RT-PCR testing on upper respiratory specimens or IgM detection on blood samples at screening.”

Correction: Clarify “screening” by specifying the time of evaluation.

Please cite the reference of the method you will use.

Please indicate which kit to use and the supplier

Line 88-89: “Blood samples are collected at multiple time points at screening (Day 0), at an optional visit to repeat IgM serology for inconclusive or negative Day 0 results (Day 7-10), and during follow-up visits on Day 14, Week 13, and Week 52.”

Comment: Clarify the purpose of the blood drive and what will be measured.

I didn't understand the reason for week 52, could I have an explanation?

Suggestion: Break down this sentence for greater clarity.

Line 90-93: “These blood samples will be tested to evaluate both humoral and cellular immune responses to endemic pathogens to measure variations in antibody diversity and antibody secreting cells (ASCs).”

Correction: Make sure the term “humoral” is understood in context.

You based your assessment of loss of immunity in children with measles on the fact of acute infection. Have you taken into account whether or not they have received one or 2 doses of measles vaccine? Because I think the length of time, they've been vaccinated can influence the immune response.

Line 94-96: “To explore how recent MeV infection may affect the child’s ability to respond to a controlled immune stimulus, all participants will receive rabies vaccine pre-exposure prophylaxis (PrEP) using Verorab inactivated rabies vaccine.”

Figure 2: I didn't understand the color differences. Please be more explicit.

Comment: The use of “pre-exposure prophylaxis” could be further explained for readers less familiar with the term.

Line 147-149: The study team will collect, to the extent possible, information on healthcare encounters for evaluation and treatment of illness during the year-long follow-up to determine if there is a difference in the number of encounters by study group

Correction: Please reword the sentence.

This is a study protocol that will be useful in research, but important points need to be clarified, such as the choice of study cohort, key questions about the vaccination status of the children in the cohort, and the fact that the latest measles vaccination coverage figures are not very high, which should be taken into account.

It should be made clear why the rabies vaccine was used and not another vaccine. The key points are not easy to find in the text, as the sentences are a little too long and therefore difficult to understand.
